# Acute Variceal Bleeding 11 Years After a Successful Mesoatrial Shunt for Budd-Chiari Syndrome: A Case Report

**DOI:** 10.7759/cureus.98673

**Published:** 2025-12-07

**Authors:** Amira Ali Elamin Babikir, Ammar Elnour, Fatimaelzahra Salih, Nathan Kevin Peter, Hussameldin Mahdi

**Affiliations:** 1 Surgery, National Health Service (NHS), London, GBR; 2 Gastroenterology, Barking, Havering and Redbridge University Hospitals National Health Service (NHS) Trust, London, GBR; 3 Acute and General Medicine, North Cumbria Integrated Care National Health Service (NHS) Foundation Trust, London, GBR; 4 Acute and General Medicine, Barking, Havering and Redbridge University Hospitals National Health Service (NHS) Trust, London, GBR; 5 Gastroenterology, Wrightington, Wigan and Leigh National Health Service (NHS) Trust, Manchester, GBR

**Keywords:** budd chiari syndrome, endoscopy, mesoatrial shunt, tipss, variceal bleeding

## Abstract

A 31-year-old male patient underwent a mesoatrial shunt surgery in July 2013, after he presented with a few months' history of recurrent abdominal pain and was subsequently diagnosed with Budd-Chiari syndrome. After the shunt surgery, he remained stable for around 11 years without any follow-up, until he presented to the hospital in May 2024 with acute variceal bleeding.

The patient presented with haematemesis, abdominal pain, and melaena. He received a blood transfusion along with resuscitation measures and medical management for suspected acute variceal bleeding. He underwent endoscopic variceal banding successfully in less than 12 hours after his presentation. Eventually, he was referred to a higher centre where he underwent a transjugular intrahepatic portosystemic shunt (TIPSS) procedure and was eventually discharged home in good condition. In conclusion, mesoatrial shunt may still be considered as a viable treatment option for Budd-Chiari syndrome, when an experienced intervention radiologist for TIPSS or liver transplant is not available.

## Introduction

Budd-Chiari syndrome (BCS) is a rare but serious vascular disorder caused by the obstruction of hepatic venous outflow, leading to impaired blood drainage from the liver. This obstruction can occur at any level, from the small hepatic veins to the junction of the inferior vena cava (IVC) with the right atrium. The resulting elevated hepatic venous pressure causes liver congestion and dysfunction, which may progress to portal hypertension, cirrhosis, and ultimately liver failure [1-3].

The aetiology of BCS is diverse and includes hypercoagulable states (e.g., polycythaemia vera, antiphospholipid syndrome, and factor V Leiden mutation), local venous thrombosis, and external compression by tumours or cysts. In some cases, the cause remains idiopathic. Clinical presentation varies widely, ranging from asymptomatic cases to fulminant hepatic failure, depending on the extent and speed of venous occlusion [3-6].

Gastro-oesophageal varices haemorrhage constitutes the most dramatic and lethal complication of cirrhosis, variceal haemorrhage. Gastro-oesophageal varices are present in approximately 50% of cirrhotic patients [7].

Prognosis is highly variable and depends on the underlying cause and the degree of liver damage at diagnosis. Early detection and appropriately tailored therapeutic interventions are essential for improving outcomes [8-11].

## Case presentation

A 31-year-old male patient presented to our hospital with a one-day history of repeated episodes of haematemesis, characterised by the vomiting of fresh blood with clots. This was associated with the passage of black, tarry stools (melaena) and generalised abdominal pain. He had a past medical history of a road traffic accident in India in 2013, which resulted in BCS. This was managed surgically with the placement of a mesoatrial shunt, and he was started on anticoagulation therapy with nicoumalone, which he had been taking regularly. However, he had not undergone any follow-up for the past six years, as he remained asymptomatic and did not present to any healthcare facility with any complaint. There was no family history of liver disease or thrombophilia.

Clinical findings

On presentation, the patient was conscious, fully oriented, and able to communicate with the medical team. His National Early Warning Score (NEWS) was 2. Vital signs included a blood pressure of 128/67 mmHg, heart rate of 133 bpm, respiratory rate of 18 breaths per minute, oxygen saturation of 96% on room air, and a temperature of 36.5°C. Physical examination revealed a soft abdomen with generalised tenderness.

Diagnostic assessment

Initial laboratory investigations are summarised in Table 1. The Glasgow-Blatchford Score (GBS) was 13. His haemoglobin on presentation was 100 g/L, which subsequently dropped to 68 g/L. A chest X-ray was unremarkable.

**Table 1:** Laboratory investigations obtained on the first day of admission and on the day of discharge are summarised above. On presentation, the patient’s haemoglobin was 100 g/L, which subsequently declined to 68 g/L. Blood urea was elevated with a normal creatinine level, findings consistent with ongoing gastrointestinal bleeding. Thrombocytopaenia and hypoalbuminaemia were also noted, supporting an underlying diagnosis of chronic liver disease. Serological testing for viral hepatitis and the autoimmune hepatitis panel were negative. PT: prothrombin time; ALP: alkaline phosphatase; ALT: alanine transaminase; ANA: antinuclear antibody; Ab: antibody; CMV: cytomegalovirus; EBV: Epstein-Barr virus

	Day 0	Day 4		
Blood test	Results	Results	Range	Units
White cell count	10.2	8.9	3.8-11.0	×10^9^/L
Haemoglobin	100	77	133-177	g/L
Platelet	146	71	150-400	×10^9^/L
PT	13.1	-	9-13	Seconds
Sodium	136	135	133-146	mmol/L
Potassium	4.8	3.4	3.5-5.3	mmol/L
Urea	11.7	5.6	2.5-7.8	mmol/L
Creatinine	53	54	59-104	μmol/L
Albumin	36	32	35-50	g/L
ALP	157	92	30-130	U/L
ALT	36	156	<41	U/L
Total bilirubin	15	10	1-21	μmol/L
Adjusted calcium	2.35	2.17	2.20-2.60	mmol/L
CRP	2	4	<5	mg/L
Ferritin		24.2	30-400	μg/L
HIV 1&2 antibody p24 antigen	Not detected	-	-	-
Hepatitis B surface antigen	Not detected	-	-	-
Hepatitis B core total antibody	Not detected	-	-	-
Hepatitis C antibody	Not detected	-	-	-
Hepatitis B surface antibody	<10 IU/mL: no response	-	-	-
Paracetamol	-	<1	-	mg/L
Serum IgG	-	9.30	5.5-16.5	g/L
Serum IgA	-	1.89	0.8-4.0	g/L
Serum IgM	-	0.95	0.5-1.90	g/L
Serum caeruloplasmin	-	0.30	0.15-0.30	g/L
ANA	-	Negative	-	-
Gastric parietal cell Ab	-	Negative	-	
Mitochondrial Ab	-	Negative	-	-
Anti-smooth muscle Abs	-	Negative	-	-
Liver/kidney micro	-	Negative	-	-
CMV IgG Ab	-	Detected	-	-
EBV VCA IgG Ab	-	Detected	-	
EBV VCA IgM Ab	-	Not detected	-	-
EBV EBNA IgG Ab	-	Detected	-	-

**Figure 1:**
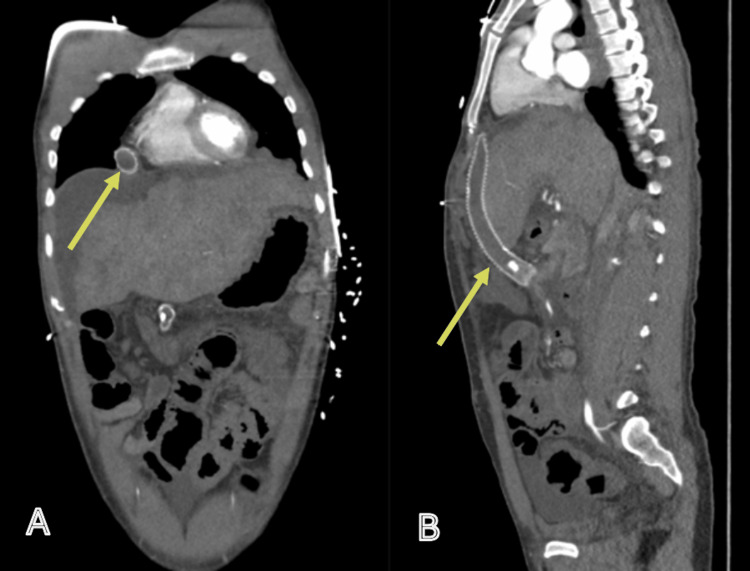
CT angiogram: arterial phase. CT angiogram in the arterial phase: coronal (A) and sagittal (B) views. The mesoatrial shunt is visualised in situ but demonstrates no contrast enhancement (indicated by the arrows). The coronal image (A) also reveals a mottled appearance of the liver parenchyma, consistent with features of cirrhosis. CT: computed tomography

**Figure 2:**
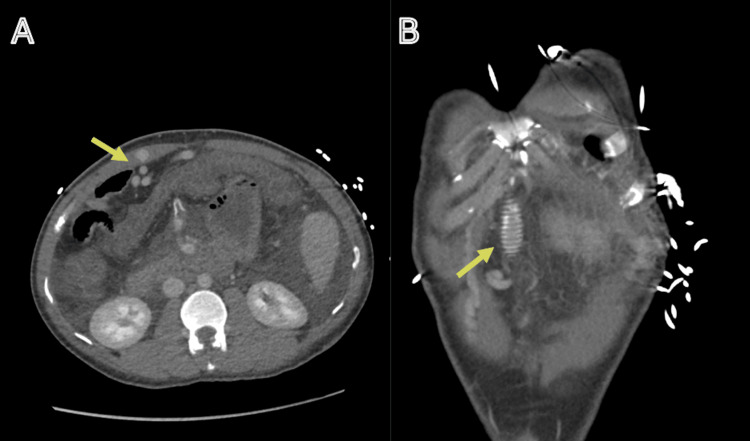
CT angiogram: portal phase. CT angiogram in the portal venous phase: axial (A) and coronal (B) views. The axial view (A) shows recanalisation of the umbilical vein (arrow), while the coronal view (B) demonstrates the presence of the mesoatrial stent (arrow). CT: computed tomography

A contrast-enhanced computed tomography (CT) scan was done (Figures 1, 2).

Imaging demonstrates the mesoatrial shunt in situ. Features are consistent with advanced chronic liver disease, including cirrhosis with portal hypertension and recanalisation of the umbilical vein. The liver parenchyma exhibits a mottled appearance, further supporting cirrhosis. Associated findings include splenomegaly and a splenic artery aneurysm. Extensive variceal formation is present in the perihepatic, perisplenic, perigastric, and para-oesophageal regions. There is moderate ascites involving the abdomen and pelvis. No active contrast extravasation is identified. There is no evidence of abdominal or pelvic lymphadenopathy. Diffuse mural thickening of the colon is noted, in keeping with colitis.

**Figure 3:**
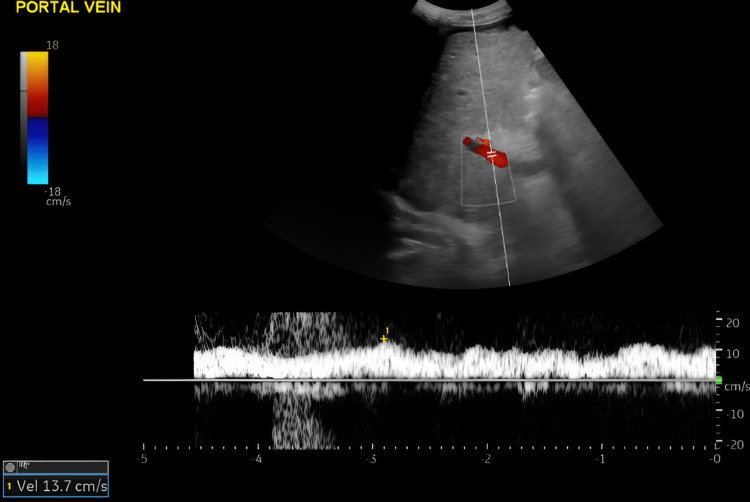
Portal and hepatic veins Doppler ultrasound. Portal and hepatic veins Doppler ultrasound showing antegrade portal venous flow, PSV = 13.7 cm/s. Hepatic veins are not visualised possibly due to the inhomogeneous appearance of the liver parenchyma. PSV: peak systolic velocity

A Doppler ultrasound of the portal and hepatic veins was carried out (Figure 3), showing an antegrade portal venous flow, peak systolic velocity (PSV) = 13.7 cm/s. Hepatic veins were not visualised due to the inhomogeneous appearance of the liver parenchyma.

Therapeutic interventions

The patient was initially managed in the Emergency Department Resuscitation (ED-Resus) area, where he received four units of packed red blood cells (PRBCs) and two units of fresh frozen plasma (FFP). He was started on a proton pump inhibitor (PPI) infusion, intravenous terlipressin, intravenous fluids for hydration, analgesia, and broad-spectrum antibiotics.

He subsequently underwent an upper gastrointestinal endoscopy, which revealed four columns of large oesophageal varices, with minor oozing from the lower varix at the gastro-oesophageal junction. Fresh blood and coffee-ground material were present in the stomach and were suctioned to improve visualisation. Four variceal bands were applied successfully.

Follow-up and outcomes

After three days of hospitalisation, the patient was transferred to a specialised centre for consideration of a transjugular intrahepatic portosystemic shunt (TIPSS) procedure, which was successfully performed five days later. Both echocardiography and electroencephalography (EEG) were normal. His anticoagulation therapy was switched to apixaban.

During follow-up in the hepatology clinic, there was no clinical evidence of ascites, and the patient reported no further episodes of haematemesis or melaena. A triple-phase liver CT scan performed in July 2024 showed an occluded mesoatrial shunt, a patent TIPSS, and progressive severe narrowing of the hepatic segment of the IVC.

## Discussion

BCS is characterised by manifestations of portal hypertension, IVC occlusion, or both. It results in liver damage due to intense venous congestion, which can ultimately lead to the development of cirrhosis [3]. Gastro-oesophageal varices haemorrhage constitutes the most dramatic and lethal complication of cirrhosis, variceal haemorrhage. Gastro-oesophageal varices are present in approximately 50% of cirrhotic patients [7].

Common symptoms include right upper quadrant abdominal pain, hepatomegaly, ascites, jaundice, and, in severe cases, gastrointestinal bleeding due to variceal rupture. Diagnosis is primarily established through imaging modalities such as Doppler ultrasound, CT scan, or magnetic resonance imaging (MRI), which reveal hepatic vein obstruction and associated complications [3,12].

Management strategies for BCS focus on restoring hepatic venous outflow, preventing further thrombosis, and treating complications. To relieve congestion, a variety of non-surgical and surgical interventions have been described [13,14].

Surgical interventions with proven survival benefit in BCS include shunt procedures and liver transplantation. Various surgical shunts can be used to decompress the liver, including mesoatrial, mesocaval, and portocaval shunts. If there is no IVC thrombosis, a side-to-side portocaval shunt is typically the procedure of choice during the early and intermediate stages of the disease [13,14].

In centres where liver transplantation or TIPSS procedures are not available, a mesoatrial shunt may be considered in carefully selected patients. This type of shunt is usually constructed from the portal-mesenteric system to the right atrium, thereby bypassing the IVC when it is occluded or when there is a significant pressure gradient. Mesoatrial shunt with a ringed polytetrafluoroethylene graft is effective in BCS cases with thrombosis or significant stenosis in the IVC [15].

Mesoatrial shunt surgery was described as a new surgical treatment in the seventies of the last century by Cameron and Maddrey [16]. The shunt requires both an abdominal and a thoracic incision. However, it can be performed synchronously by two surgical teams and thus completed in less time than a standard portosystemic shunt.

If the IVC is occluded or has elevated pressure, a mesoatrial shunt should be performed. This approach should result in decompression of the engorged liver with preservation of hepatocyte function and could greatly increase survival in patients with BCS [13-15].

Only a few cases were reported after a successful mesoatrial shunt. For example, there is a case report of an eight-year follow-up [17]. Another report mentioned an excellent outcome six years after the establishment of a mesocavoatrial shunt where two grafts were placed, therefore concluding that the mesocavoatrial shunt could have a satisfactory long-term patency. Thus, the use of mesocavoatrial shunts for treating patients with severe BCS without the possibility of medical therapy and intervention is suggested [18].

Zhu et al. [19] described the long-term efficacy by retrospectively evaluating 11 patients who underwent mesoatrial shunt for BCS. The results showed that 63.3% of patients had survived for more than 10 years and 45.5% for more than 20 years. A man has been alive with a patent shunt for 28 years and one month. These indicate that a mesoatrial shunt is an effective therapeutic modality for appropriate patients with BCS (suprahepatic IVC occlusion, or patients with long segmental occlusion of IVC involving hepatic veins, or suprahepatic IVC occlusion that failed to respond to interventional procedures). Meanwhile, good hepatic function (Child-Pugh class A, B) is necessary for patients to tolerate the mesoatrial shunt and recover postoperatively [19].

Another study of 10 patients reported a median follow-up of 40 months [20]. However, the long-term efficacy of this surgery was not well studied.

## Conclusions

This case is notable for the exceptional longevity of the mesoatrial shunt, which remained patent for approximately 11 years, during which the patient remained asymptomatic. It is also unique in demonstrating successful acute management of variceal bleeding through endoscopic variceal band ligation, followed shortly by a successful TIPSS procedure. This combined approach resulted in the complete resolution of ascites and a relatively short hospital stay.

Further retrospective studies are warranted to assess the long-term efficacy of the mesoatrial shunt procedure. However, with the increasing availability of specialised liver centres and the growing use of TIPSS-which has proven to be both highly effective and less invasive-accumulating long-term data on surgical shunts may be challenging. Indeed, TIPSS has now largely supplanted traditional surgical shunting techniques. While this case highlights that mesoatrial shunts can remain functional for many years, the relevance of this procedure in current practice is limited and should be interpreted with caution. Clinicians should consider contemporary alternatives, such as TIPSS, while recognising that rare patients may still benefit from surgical shunting in selected circumstances.
